# Evaluation of the Shear Bond Strength of Chitosan Nanoparticles-Containing Orthodontic Primer: An In Vitro Study

**DOI:** 10.1155/2023/9246297

**Published:** 2023-08-03

**Authors:** Rawnaq R. Mohammed, Reem A. Rafeeq

**Affiliations:** ^1^Master Student, Department of Orthodontics, College of Dentistry, University of Baghdad, Baghdad, Iraq; ^2^Al Najaf Health Directorate, Ministry of Health, Najaf, Iraq; ^3^Professor, Department of Orthodontics, College of Dentistry, University of Baghdad, Baghdad, Iraq

## Abstract

**Objectives:**

The present study was intended to investigate the effect of different concentrations of chitosan nanoparticles mixed with an orthodontic primer on the shear bond strength and bond failure of stainless steel brackets bonded to dental enamel.

**Methods:**

Four concentrations of chitosan nanoparticles (0%, 1%, 5%, and 10%) were prepared and mixed with Transbond™ XT primer. Forty-eight extracted maxillary first premolars were bonded under a standardized procedure with stainless steel orthodontic brackets utilizing those different concentrations (12 teeth per each group). After the bonding procedure, the specimens were stored in deionized water (37°C for 24 hr) and then thermocycling 5,000 times before shear bond testing, which was performed using a universal testing device. Bond failure sites were examined under a stereomicroscope. Scanning electron microscopy and X-ray diffraction were also performed to verify and evaluate the phase of the nanopowder.

**Results:**

The data were statistically analyzed using one-way analysis of variance) and Kruskal–Wallis *H* tests, and the findings revealed statistically nonsignificant group differences regarding the shear bond strength and adhesive remnant index (*p* > 0.05).

**Conclusions:**

Primers containing varying concentrations of chitosan nanoparticles demonstrated acceptable shear bonding strength and adhesive remnant index.

## 1. Introduction

White spot lesions (WSLs) are enamel demineralization patches resulting from fixed orthodontic appliance treatment which occur along the contour of the bracket base [1]. The prevalence is commonly stated to be between 25% and 30%; however, this varies depending on the detection criteria used and the individual's overall caries risk, with a high rate after 12 months of orthodontic equipment with no group or gender differences [2]. Many materials and treatments are utilized to reduce bacterial colonization and improve remineralization, however negative effects limit their use [3–5]. Many scientists are investigating the use of fluoride in adhesives to promote remineralization [6]. Unfortunately, the effect of these additives may last only a few weeks, resulting in greater adhesive failure rates [7]. Other researchers investigated the inclusion of metal nanoparticles in adhesives in order to maximize the use of nanotechnology properties; however, these modifications may impair shear bond strength [8], and the addition of certain metal nanoparticles into orthodontic resin causes unfavorable changes in enamel color [9]. The reduced bracket bond strength during orthodontic treatment may increase the chance of bracket debonding and, as a result, treatment time and patient frustration [10]. Furthermore, the use of metal nanoparticles increases cytotoxicity [11]. Chitosan is a naturally produced biopolymer which is created via deacetylation of chitin [12]. Chitosan and its derivatives have piqued the interest of researchers due to its ability to reduce *Streptococcus mutans*, *Streptococcus sanguinis*, and fungi [13]. This polysaccharide-based polymer offers nontoxic, biodegradable and biocompatible properties that can be used to replace the limits of metal nanoparticles [14]. The goals of this study are to develop a chitosan-containing orthodontic primer and examine how incorporating chitosan nanoparticles to orthodontic primer impacts the shear bond strength and bond failure of stainless steel brackets adhered to tooth enamel. The present study is the first to develop a novel orthodontic primer and examine the shear bond of an orthodontic primer incorporating chitosan nanoparticles.

## 2. Materials and Methods

### 2.1. Nanoparticles Characterization

Chitosan nanopowder with a purity of 99%, a size of 50 nm, and a crystalline shape were created by the Nano Materials Iranian Company (Nano materials Iranian company, Tehran, Iran), and the phase of the nanopowder was confirmed and evaluated using an X-ray diffraction (XRD) equipment (PHILIPS_PW1730, Netherlands). Also the nanoparticle was examined by field-emission scanning electron microscopy (SEM) using TESCAN MIRA (Mira3-XMU model – Brisbane, Queensland, Australia).

### 2.2. Preparation of Orthodontic Primer and Sample Preparation

A digital electronic scale (precision of 0.001 g) (Satorius Company, Göttingen, Germany) was used to weigh chitosan nanoparticles and Transbond^TM^ XT orthodontic primer (3M-Unitek, Monrovia, USA). Chitosan nanoparticles were added to Transbond^TM^ XT primer in 0%, 1%, 5%, and 10% concentrations by using each drop weighed at 0.05 g, so to prepare 1 g of primer, and use 20 drops for each group, which were mixed with a different concentration of chitosan (0.01, 0.05, and 0.1 g), and for the control group without any addition. After that, a vortex mixer (Stuart Scientific, England, UK) was used for 2 min to obtain optimum homogeneity. The standardization of drops is achieved by using a 10–100 *µ*m sampler (Cypress Diagnostics, Hulshout, Belgium). The addition and mixing procedures were conducted in a semidark room [13].

**Figure 1:**
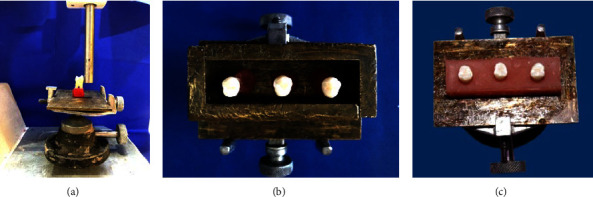
Construction of acrylic blocks. Standardizing the inclination of the teeth (a), a mold made to mount the teeth after coating with petroleum jelly (b), and final adjustment of the acrylic block (c).

Forty-eight extracted human upper premolar teeth were chosen and preserved in a 0.2% (w/v) thymol solution that was changed weekly [15]. First, the extracted premolars were examined using a magnifying lens (10x) (Citoglas, Jiangsu, China) to confirm that the buccal enamel surface lacked cavities, fractures, and hypoplastic areas. Then, the teeth were standardized and mounted in cold-cure acrylic (Veracril®, Guarne, Colombia) [16], and until the bonding step, the blocks were held in deionized water [17], as illustrated in Figure 1.

Transbond™ XT orthodontic adhesive (3M-Unitek, Monrovia, USA) was used to bond the Discovery bracket (Dentaurum company, Ispringen, Germany) to the teeth. According to the manufacturer's recommendations, the buccal surface was etched for 30 s with 37% phosphoric acid (SDI, California, USA), then rinsed with water for 20 s before being dried for 10 s to produce an ice-white surface [18]. Then primer was applied to the tooth using an applicator (3M Unitek, Monrovia, USA), softly distributed with air, and light-cured for 10 s [19]. After that, an equal amount of the Transbond™ XT adhesive was applied to the base of the brackets and then bonded into the middle third of the buccal surface by using a dental surveyor (Paraline, Dentaurum, Pforzheim, Germany) [16].

**Figure 2:**
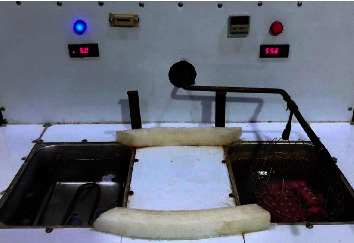
Thermocycling of 5,000 cycles between 5 and 55°C.

After bonding, the samples were stored in deionized water at 37°C in an incubator (Binder, Series BD-S Solid Line, Tuttlingen, Germany). They were subsequently handled for 5,000 cycles of hot- and cold-water baths (5 and 55°C, respectively), with a 30 s duration in each bath and a 5 s transfer delay between each [20]. Following thermocycling, the samples were kept at room temperature in deionized water until the shear bond strength test [16], as shown in Figure 2.

**Figure 3:**
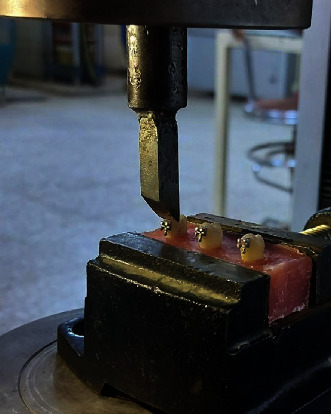
Fixation of the sample in the universal testing machine.

The SBS test was carried out using a Tinius–Olsen universal testing instrument (Instron machine, model: wdw50, Laryee Technology, Beijing, China) equipped with a 50 kN load cell and a crosshead speed of 1 mm/min [21]. First, each specimen was clamped in the testing machine's lower jaw with a unique clamping tool before the chisel's pointed end was placed in the upper arm with its side parallel to the bonding surfaces. Then, at the tube base/bonding surface interface, a shearing force was applied in an occlusal–gingival direction until debonding happened [16], as shown in Figure 3.

A stereomicroscope (Leica™, Leitz, Wetzlar, Germany) was used to evaluate the bond failure site [22]. Following debonding, the bonding surfaces and base of the bracket were evaluated for adhesive remnant index (ARI) utilizing Artun and Bergland's 1984 classification, which created the ARI method to measure the quantity of adhesive left on the tooth surface after debonding with a score ranging from 0 to 3 which is one of the most frequently employed indices in the orthodontic investigation [23]. Hellak et al. [24] produced a modified version of this approach, which was employed in the current investigation since it includes an additional score for surface fracture.

### 2.3. Sample Grouping

Using G power 3.1.9.7 (Program written by Franz-Faul, Universitat Kiel, Germany) with the power of study = 80%, alpha error of probability = 0.05 two-sided, doing a pilot study for three samples for each group found that the highest mean is 23.50 and the lowest mean is 18, and the pooled standard deviation is 5 thus the effect size of *F* is 0.50 (large effect size), with four groups, with all these conditions the sample size 48 samples (12 samples for each group).

The groupings were as follows:

- Group A: this group, represents the control group bonding with primer without additives.
- Group B: this group, bonding with primer, has 1% chitosan nanoparticles.
- Group C: this group, bonding with primer, has 5% chitosan nanoparticles.
- Group D: this group, bonding with primer, has 10% chitosan nanoparticles.

### 2.4. Statistical Analysis

The statistical analysis of the data was performed using SPSS Statistics™ software version 25 (Statistical Package of Social Sciences) (IBM Company, New York, USA). The Shapiro–Wilk test was used to test the normality of the data distribution. Next, a one-way ANOVA test was used to determine whether there were any statistically significant differences between measured SBS values among different groups. The Kruskal–Wallis test was used to determine whether there are any statistically significant differences between measured ARI scores among different groups. Finally, post hoc tests assessed the differences between groups if the ANOVA test revealed significant differences.

## 3. Result

### 3.1. X-Ray Diffraction

**Figure 4:**
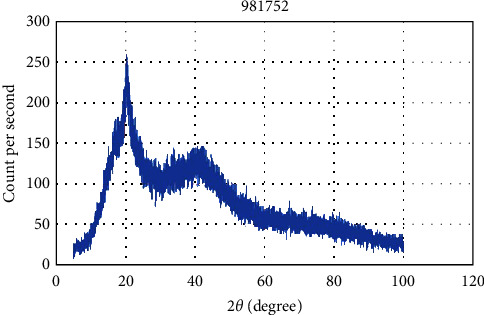
XRD patterns of chitosan nanoparticles.

Figure 4 depicts the X-ray diffractograms of Chitosan nanopowder. Six diffraction peaks were found at 2 = 5.900, 9.000, 10.320, 16.970, 20.100, and 40.730. The prepared sample's average crystallite size was around 21.48 nm.

### 3.2. Scanning Electron Microscope Analysis

**Figure 5:**
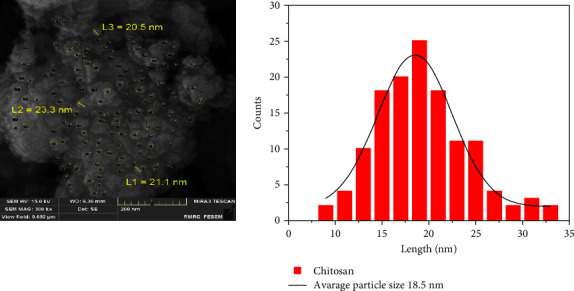
SEM image and histogram distribution of chitosan nanoparticles.

A scanning electron microscope was used to examine the surface morphology of chitosan nanoparticles and to determine the shape and average dimensions of the atoms. The results indicate that the shape of most of the nanoparticles is almost spherical, with a diameter ranging between 6 and 33 nm, as shown in Figure 5.

The average atomic dimensions and atomic distribution histogram were calculated using the modern programmers ImageJ (Wayne Rasband and contributors, National Institutes of Health, USA) and Origin Pro (OriginLab Corporation, Northampton, MA 01060, USA), respectively. The analytical results of the electron microscope images showed that the average dimensions of the atoms are 18.5 nm.

### 3.3. Shear Bond Strength

#### 3.3.1. The Normality of Distribution of SBS Data

The Shapiro–Wilk test was used to see if the data followed a normal distribution. All groups show a *p*-value greater than 0.05, so the data is nonsignificant. Depending on that, accept H0, which means the data follow a normal distribution (*p* > 0.05) as shown in Table 1.

#### 3.3.2. The Test for Variance Homogeneity

Levene's test was performed to look at the consistency of the data, and it showed that there was not much of a distinction between the groups (*p* > 0.05).

#### 3.3.3. SBS Descriptive Statistics

The mean SBS, standard deviation, minimum, and maximum values for each group are shown in Table 2. The 1% group had the highest mean SBS value (21.07 ± 5.52 MPa), followed by the 5% group (20.82 ± 4.39 MPa), then the 10% group (19.01 ± 6.10 MPa), and finally the control group (18.09 ± 5.28 MPa). In addition, there were nonstatistically significant differences after comparing the mean SBS differences between the groups using a one-way ANOVA test.

### 3.4. Adhesive Remnant Index

**Figure 6:**
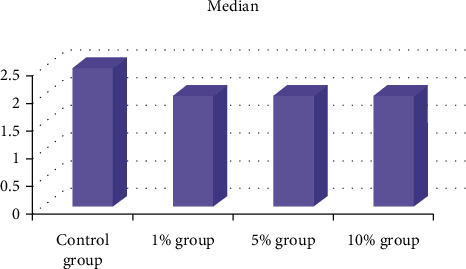
Median of ARI for the control group and other groups.

The control group had the highest ARI median score, while the other groups had the lowest (Table 3 and Figure 6).The Kruskal–Wallis test, on the other hand, found no statistically significant differences between the four groups (Table 4).

## 4. Discussion

WSL is a typical lesion during orthodontic treatment, compromises enamel integrity, lasts a long time, and is challenging to restore through natural remineralization completely [25]. Numerous studies found that nanoparticle-containing primers successfully reduced demineralization scores within the first 6 months following bracket bonding [26]. Meanwhile, attempts to add nanoparticles to the adhesive solution, such as silver, zinc oxide, gold, hydroxyapatite, and titanium dioxide, harmed mechanical properties [27]. Nevertheless, none of the earlier studies looked at how chitosan nanoparticles may affect the mechanical properties of orthodontic primers. The present study is the first to develop a novel orthodontic primer and examine the shear bond of an orthodontic primer incorporating chitosan nanoparticles. Therefore, chitosan nanoparticles were chosen for their chemical stability, availability, biocompatibility, and antibacterial activity [28]. The results from the XRD investigations in the present study revealed that a sharp and high-crystalline peak is observed at the angle position 2*θ* = 20.10°; this crystalline peak at an angle (20.10°) of pure chitosan can be attributed to the Miller plate at the plane (220) [29]. The crystalline structure of chitosan is due to hydrogen bonds within the molecule, and there is a broad peak that extends from 35° to 55°; it indicates the amorphous region of chitosan, consistent with previous studies [30]. A scanning electron microscope revealed that small particle dimensions are due to the ionic generation method for preparing chitosan, which is widely used in preparing chitosan nanoparticles. The chitosan preparation technology provides rapid production with small nanoparticles. Previous studies indicate the possibility of controlling and modifying the surface and size of nanoparticles by changing the proportions of chitosan and stabilizer [31]. The chosen sample was human maxillary premolars, and the results may be verified in clinical practice [32].

Reynolds [33] proposed a minimum clinical range for shear bond strength of 6–8 MPa. The current study shows that incorporating 1% chitosan nanoparticles increases the orthodontic shear bond strength; however, increasing the percentage of nanoparticles to more than 1% reduces the bond strength but still exceeds the minimum limit determined by Reynolds [33] and no statistically significant differences in SBS mean values among groups in this study, demonstrating that all primers have adequate resistant to shear stress and had no discernible impact on the SBS of the tested primers, which is agreed to the previous study's findings [34, 35]. Therefore, an increase in the shear bond strength of 1% chitosan nanoparticles containing primer has been offered as evidence that nanoparticles work as stress-absorbing materials that provide structural reinforcement. As a consequence of this, it helps reduce interfacial stress concentration within the adhesion–resin complex.

Moreover, employing a vortex to mix the nanoparticles with the primer in the current study, rather than hand mixing, may have contributed to the nanoparticles dispersing correctly and, as a result, agglomeration not forming. The initially observed increase in shear bond strength is consistent with previous findings [36, 37]. Even at 10% chitosan nanoparticle concentrations, there was no significant change in mechanical properties compared to the control group in the current study. The result might be caused by the nanomaterial's small size of 50 nm, which makes distribution easier within the primer, which has a low viscosity. While the other studies applied nanoparticles to composites, they influenced the SBS because the compact nature of the adhesive may disrupt the sticky matrix; this also may be due to differences in the methodology involving the type of nanoparticles, concentration, size, and methods of adding the nanoparticles [38].

Environmental variables such as temperature, humidity, and intraoral contaminants can all affect clinical bonding strength, therefore, depending on the temperature, although many manufacturers advise keeping dental adhesive systems in the refrigerator. Previous research discovered that the lowest bond strength values were obtained at 4°C because adhesive viscosity increases significantly at low temperatures, and if the adhesive systems are heated to 55°C, some adhesive components, such as HEMA, MDP, and BIS-GMA, may have chemically degraded; however, the SBS values found to be appropriate when the adhesive systems heated to 36°C [39]. Another consideration is that in vitro thermocycling is frequently utilized to simulate temperature and moisture variations in the oral environment to assess their impact on shear strength [40]. Hence, several studies found statistically significant alterations in the presence of thermocycling because the constant action of water in hydrolytic breakdown may induce a reduction in shear bond strength [41]. In the present study, the topic of cycling is not variable and applies to all groups. Earlier research discovered that saliva contamination affects shear bond strength and that using a conventional hydrophobic bonding system in a wet environment produced a predominantly adhesive fracture pattern; this could be explained by salivary protein penetration into microretentions formed in the tooth enamel, reducing its adhesive capacity [42]. Blood contamination, on the other hand, minimized the SBS value considerably more compared to saliva contamination due to proteins in blood clots that interfere with the penetration of adhesive substances into the enamel, and both conventional and hydrophilic primers generated significant reductions in bond strengths in blood-contaminated conditions [43]. Also, bleaching immediately before bonding and 1 week before bonding decreased the bond strength of the adhesive, and the authors interpreted this finding as follows: the bleaching agents released free radicals as nascent oxygen and hydroxyl or peri-hydroxyl ions when they applied to the dental structure, and this property may be detrimental to resinous material bonding by interfering with the polymerization process of the adhesive materials [44]. While bleached enamel surface pretreatment with Er:YAG and CO_2_ lasers will enhance SBS because the etching of enamel surfaces by laser causes surface irregularity that provides the required bonding surface [45]. Some studies found that laser treatment of dental enamel causes a thermally induced process due to the vaporization of the water trapped within a depth of 10–20 *µ*m of the enamel hydroxyapatite matrix; however, more consistent enamel surface alterations and SBS values without any thermal damage were demonstrated in more lately studies [46].

In the current study, no significant difference was found in the ARI distribution on the surface of the teeth among the different groups. This finding is consistent with the findings of previous studies [47, 48]. While there was disagreement with a study that found a significant difference between the control and nanoparticle groups [8], this could be attributed to the NPs utilized in their investigation having rather large particle sizes, causing an impediment to adhesive deposition into microporosities on the tooth surface. Most of the samples had scores I in the 1%, 10%, and 5% groups, respectively, whereas the control group scored III. Therefore, less than 50% of the adhesive substance remained on the tooth surface (score I), which was advantageous for time-saving purposes, and other studies state that this score is favorable since chair time would be decreased [49]. Different studies have been conducted after adding calcium fluoride nanoparticles to the orthodontic primer [27], copper nanoparticles to orthodontic composite [35], and titanium oxide nanoparticles to orthodontic composite [37], which may be an alternative treatment nanoparticle for the current study.

## 5. Clinical Implications

Biochemical and mechanical features of chitosan-containing primer candidates are to be investigated in the clinical environment to reduce the incidence of WSLs. Incorporating nanoparticles into the orthodontic primer rather than the composite adhesive may be more effective. Because of the primer's low viscosity, it will come into contact directly with the enamel.

Furthermore, the nanoparticles can penetrate deep enough as the acid etch conditions the enamel surface and, as a result, develops the outer enamel layer against acid violence. Further studies are needed to investigate (a) if these in vitro results can be confirmed under in vivo conditions and (b) antibacterial activity tests against *S. mutans* bacteria can be carried out to assess the antibacterial activity of chitosan nanoparticle-containing orthodontic primer.

## 6. Conclusion

The use of nanoparticles in dentistry is rapidly evolving; however, in vivo, the application of these materials must be demonstrated to be feasible. This research employed chitosan nanoparticles with an approximate size of 50 nm. Orthodontic primers containing varied concentrations of chitosan nanoparticles (1%, 5%, and 10%) demonstrated appropriate SBS and ARI.

## Figures and Tables

**Table 1:** Shapiro–Wilk's normality testing.

Group	Statistics	d*f*	*p*
Control group	0.927	12	0.352
1% group	0.885	12	0.101
5% group	0.957	12	0.738
10% group	0.921	12	0.297

**Table 2:** Descriptive and comparative statistics of SBS (MPa) values.

Groups	*N*	Mean	Standard deviation	Minimum	Maximum	*F*	Sig.
Control group	12	18.0983	5.28104	8.90	26.69	0.861	0.469
1% group	12	21.0742	5.52812	14.69	30.33		
5% group	12	20.8275	4.39832	14.01	27.50		
10% group	12	19.0100	6.10654	8.73	30.53		

**Table 3:** ARI descriptive statistics.

Group	*N*	Median	Mean rank	Min.	Max.
Control group	12	2.5	28.67	0	4
1% group	12	2	21.29	0	4
5% group	12	2	26.75	1	4
10% group	12	2	21.29	0	4

**Table 4:** Kruskal–Wallis test for ARI with different primers.

	ARI
Kruskal–Wallis *H*	2.946
d*f*	3.000
Asymp. sig.	0.400

## Data Availability

This article contains the data that support the study's results.

## Additional Points

*Limitations*. Similar to other in vitro studies, this study does not entirely reflect the intraoral environment. Concerning ARI, the results should be explained carefully due to the small sample size and large variability. However, incorporating chitosan nanoparticles in both the prime and composite were not possible for this in vitro study as this required a specified manufacturing technique that restricted this combination.
